# Differential protein expression during growth on linear versus branched alkanes in the obligate marine hydrocarbon‐degrading bacterium *Alcanivorax borkumensis* SK2^T^

**DOI:** 10.1111/1462-2920.14620

**Published:** 2019-04-21

**Authors:** Benjamin H. Gregson, Gergana Metodieva, Metodi V. Metodiev, Boyd A. McKew

**Affiliations:** ^1^ School of Biological Sciences University of Essex Colchester, Essex, CO4 3SQ UK

## Abstract

*Alcanivorax borkumensis* SK2^T^ is an important obligate hydrocarbonoclastic bacterium (OHCB) that can dominate microbial communities following marine oil spills. It possesses the ability to degrade branched alkanes which provides it a competitive advantage over many other marine alkane degraders that can only degrade linear alkanes. We used LC–MS/MS shotgun proteomics to identify proteins involved in aerobic alkane degradation during growth on linear (*n*‐C_14_) or branched (pristane) alkanes. During growth on *n*‐C_14_, *A. borkumensis* expressed a complete pathway for the terminal oxidation of *n*‐alkanes to their corresponding acyl‐CoA derivatives including AlkB and AlmA, two CYP153 cytochrome P450s, an alcohol dehydrogenase and an aldehyde dehydrogenase. In contrast, during growth on pristane, an alternative alkane degradation pathway was expressed including a different cytochrome P450, an alcohol oxidase and an alcohol dehydrogenase. *A. borkumensis* also expressed a different set of enzymes for β‐oxidation of the resultant fatty acids depending on the growth substrate utilized. This study significantly enhances our understanding of the fundamental physiology of *A. borkumensis* SK2^T^ by identifying the key enzymes expressed and involved in terminal oxidation of both linear and branched alkanes. It has also highlights the differential expression of sets of β‐oxidation proteins to overcome steric hinderance from branched substrates.

## Introduction

*A. borkumensis* SK2^T^ is a model strain of a group of organisms known as obligate hydrocarbonoclastic bacteria (OHCB) which grow on a highly restricted spectrum of substrates, predominantly alkanes and their derivatives, with carbon chain length from *n‐*C_9_ to at least *n*‐C_32_ (Schneiker *et al.,* 2006; Yakimov *et al.,* 1998; 2007; Naether *et al.,* 2013). *Alcanivorax* spp. are found in low abundances in unpolluted marine environments but can multiply and grow rapidly in oil‐polluted waters, where they can constitute 80%–90% of the microbial community (Harayama *et al.,* 1999; Kasai *et al*., 2002; Syutsubo *et al.,* 2001). Previous reports have also shown *Alcanivorax* spp. become abundant in field and mesocosm experiments involving the addition of nitrogen and phosphorus fertilizers to stimulate microbial degradation of oil (Cappello *et al.,* 2007; McKew *et al.,* 2007).

*Alcanivorax* spp. has a cosmopolitan distribution with isolations or detection of its 16S rRNA gene sequences from many different oil‐impacted marine environments (Yakimov *et al.,* 2007). *Alcanivorax borkumensis* SK2^T^ (DSM 11573) was the first OHCB to have its genome sequenced revealing a plethora of genes accounting for its wide hydrocarbon substrate range and efficient oil‐degradation capabilities (Reva et al., 2008; Schneiker *et al.,* 2006). Analysis revealed proteins required to oxidize *n*‐alkanes up to the corresponding acyl‐CoA derivative are coded by the *alk*SB_1_GJH operon which has >80% amino acid similarity to the corresponding well‐characterized alkane degradation components in *Pseudomonas putida* Gpo1 (Kok *et al.,* 1989; van Beilen *et al.,* 2001; van Beilen *et al.,* 2003; van Beilen *et al.,* 2004). However, genomes only give insight into the genetic potential of an organism. Several transcriptomic studies hint even more at this potential. A microarray‐based study led to the identification of up‐regulated genes predicted to be involved in the terminal oxidation of *n*‐hexadecane (Sabirova *et al*., 2011). These included genes coding for two alkanes monooxygenases (*alkB1* and *alkB2*), three flavin‐binding monooxygenases (ABO_0282, ABO_1097, ABO_2107), an alcohol dehydrogenase (ABO_2483) and an aldehyde reductase (*AKR1A1*/ABO_2414). A subsequent microarray analysis identified genes that distinctly respond to solvent stress by the addition of 1‐octanol, a known toxic intermediate of *n*‐alkane degradation (Naether *et al.,* 2013). In accordance with Sabirova *et al*., 2011, the expression level of *alkB1* (ABO_2707) was increased during growth on the *n‐*hexadecane. In contrast, RNA‐sequencing surprisingly showed that only 1% of genes were up‐regulated while *A.borkumensis* was growing on *n*‐dodecane and none of these were involved in alkane metabolism (Barbato *et al*., 2016). The mRNA abundances presented in such studies may not directly correlate to the amount of protein within a cell, due to many factors. Large changes in gene expression may not necessarily result in similar changes in translated proteins as there can be a requirement for gene co‐regulation, controls on post‐translational modification or protein degradation, or protein translation may be highly dependent on translation initiation features and recruitment of ribosomes (Vogel and Marcotte, 2012). The proteins are the functional molecules in cells and it is the proteome, rather than the genome or transcriptome, that is, therefore most related to the phenotype of an organism, yet there is little knowledge or quantification of protein biosynthesis in *Alcanivorax* during alkane degradation. A previous study of *A.borkumensis* using two‐dimensional gel electrophoresis identified up‐regulation of two alkane hydroxylases AlkB1 and AlkB2 (Sabirova *et al.,* 2006), which have been shown to oxidize medium‐chain alkanes in the range of *n*‐C_5_‐*n‐*C_12_ and *n*‐C_8_‐*n*‐C_16_ (van Beilen *et al.,* 2004), respectively, three P450 cytochromes, and a flavin‐binding monooxygenase with a strong similarity to the AlmA enzyme, which hydroxylates long‐chain alkanes (*n‐*C_22_‐*n*‐C_36_) in *A. dieselolei* and *A. hongdengensis* (Liu *et al.,* 2011; Wang and Shao, 2012b, Wang and Shao, 2014). A downside of this proteomic technique is that it is only semiquantitative and often a relatively small number of proteins are detected (e.g. only 97 proteins were excised, sequenced and identified in Sabirova *et al.,* 2006.

The ubiquity of *Alancaivorax* and its success as an alkane degrader may result from the ability to also efficiently utilize branched alkanes as the sources of carbon, which provides a competitive advantage over other OHCB which are unable to utilize these more difficult to degrade substrates (Pirnik *et al.,* 1974; Hara *et al.,* 2003). In the previous reports, branched alkanes, such as pristane and phytane, which are the abundant branched alkanes present in many crude oils, induce the expression of AlkB1 and the long‐chain alkane monooxygenase AlmA in *A. hongdengensis* A‐11‐3 and *A. dieselolei* B‐5 (Liu *et al.,* 2011; Wang and Shao, 2012b). Sevilla *et al.,* 2017 demonstrated through transcriptional fusions of the three cytochrome P450 genes from *A. borkumensis* to a green fluorescent protein reporter gene and fluorescence assays that expression of P450‐3 was higher on pristane compared to *n‐*alkanes or pyruvate. However our knowledge of the proteins involved branched alkane degradation remains very limited. This study is first to compare the differences in protein expression during linear and branched alkane degradation in *A. borkumensis* SK2^T^. It provides insights into the metabolic pathways in this environmentally relevant hydrocarbon degrader.

We quantified changes in the proteome using liquid chromatography–tandem mass spectrometry (LC–MS/MS) identifying proteins significantly up‐regulated, while growing on either linear alkanes (tetradecane, *n*‐C_14_) or pristane (the branched alkane 2,6,10,14‐Tetramethylpentadecane) looking at the key differences in the expression patterns on each substrate.

## Results

### *Overview of LC–MS/MS shotgun proteomic analysis*

Nine LC–MS/MS runs were performed consisting of three independent biological replicates of three treatments (linear alkane (*n*‐C_14_), branched chain alkane (pristane), non‐hydrocarbon control (pyruvate)) resulting in 112,095 spectral counts that were assigned to 1309 proteins, representing 48% of the total protein‐coding genes on the *A. borkumensis* SK2^T^ genome (Supporting Information Table S1). Over half (52%) of the spectral counts were assigned to the 100 most abundantly detected proteins, and 90% were assigned to the 500 most abundant proteins. The remaining 809 proteins (representing 7% of the total normalized spectral counts) were detected with only a very low number of spectral counts.

**Figure 1:**
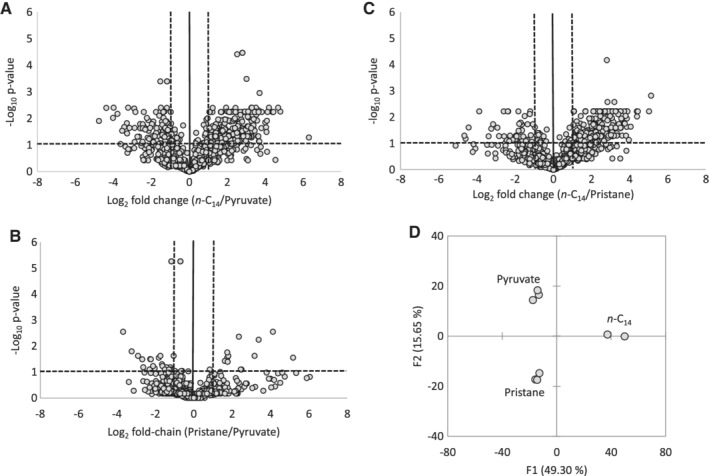
A‐C. Volcano plots of normalized LC–MS/MS spectral counts comparing *A. borkumensis* protein expression during growth on a linear alkane (*n*‐C_14_), a branched alkane (Pristane) and a non‐hydrocarbon control (Pyruvate). Data points above horizontal dashed line represent *P*‐values below 0.05. Vertical dashed lines represent a two‐fold change. D. Principle component analysis of replicate *A. borkumensis* proteomes (including normalized spectral counts of 1309 proteins) on growth substrates *n*‐C_14_, pristane and pyruvate.

A total of 381 proteins were significantly differentially expressed during growth on *n‐*C_14_ compared to pyruvate, with 80% of these being up‐regulated on *n*‐C_14_ (Fig. 1A). In contrast, only 23 proteins were differentially expressed during growth on pristane compared to pyruvate, 39% of which were up‐regulated on pristane (Fig. 1B). Between the linear (*n*‐C_14_) and branched (pristane) chain alkanes, 288 proteins were significantly differentially expressed, with 91% of these being up‐regulated on *n‐*C_14_ (Fig. 1C). Overall the total proteomes differed markedly between the three growth substrates with highly similar proteomes between replicates (Fig. 1D).

### *Terminal oxidation of linear* n*‐alkanes*

**Figure 2:**
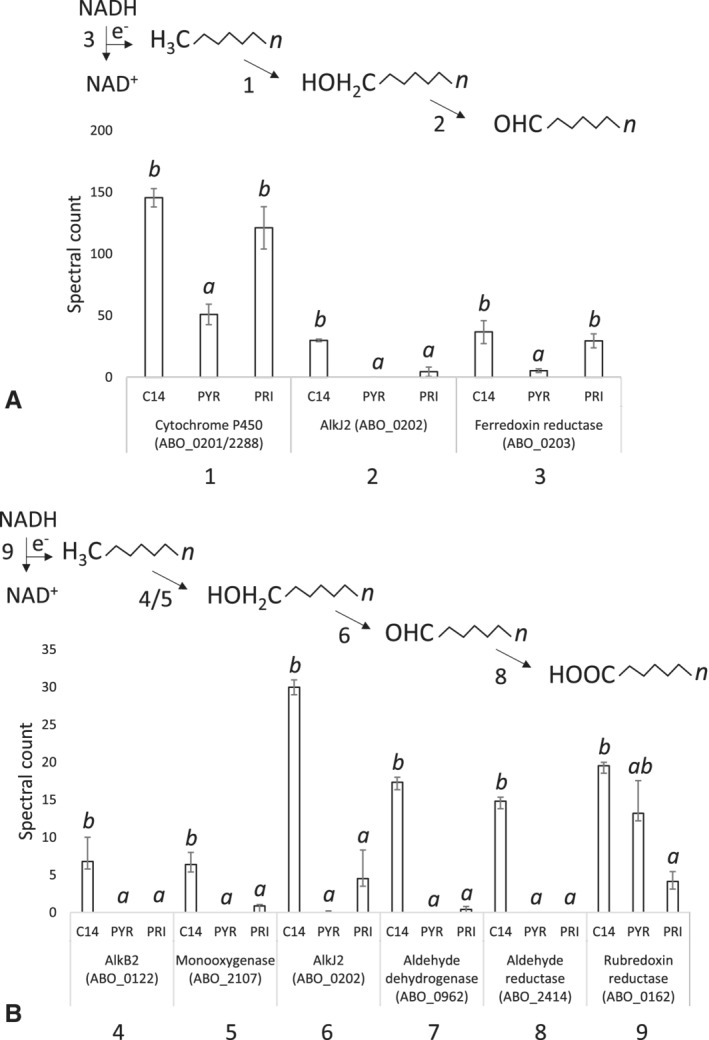
Normalized spectral counts (means ± SE; *n* = 3) of differentially expressed linear alkane oxidation proteins during growth on a linear alkane (*n*‐C_14_/C14) a non‐hydrocarbon control (Pyruvate/PYR) and a branched alkane (Pristane/PRI) in *A. borkumensis* SK2^T^; treatments not sharing a letter (*a* or *b*) differ at *P* < 0.05 (Tukey's HSD). Two different alkane oxidation systems were detected. A. 1. Cytochrome P450 introduces oxygen into the alkane at the terminal carbon converting it into a primary alcohol. 2. AlkJ2 further oxidizes the primary alcohol generated into an aldehyde. 3. Ferredoxin reductase oxidizes NADH to NAD^+^ shuttling electrons (e^−^) to the P450 through a ferredoxin. B. 4/5. Monooxygenases introduce oxygen into the alkane at the terminal carbon converting it into a primary alcohol. 6. AlkJ2 further oxidizes the primary alcohol generated into an aldehyde. 7. Aldehyde dehydrogenase converts the aldehyde into a fatty acid, which enters β‐oxidation. 8. Aldehyde reductase also converts the aldehyde into a fatty acid, which enters β‐oxidation. 9. Rubredoxin reductase oxidizes NADH to NAD^+^ shuttling electrons (e^−^) to the monooxygenase through a rubredoxin.

Several proteins involved in terminal oxidation of linear *n*‐alkanes were significantly differentially expressed during growth on *n*‐C_14_ compared to pyruvate (Fig. 2). The expression of two P450 CYP153 cytochromes (ABO_0201/2288, *P =* 0.028), which share the same amino acid (100% similarity) and their corresponding genes share 99.6% nucleotide identity, was three‐fold and two‐fold greater while growing on *n‐*C_14_ and pristane, respectively (Fig. 2A). This suggests the proteins may have a broad substrate specificity being able to utilize both linear and branched alkanes or is constitutively expressed due to expression also on pyruvate. The cytochrome P450s are part of a putative operon made up of genes coding for a ferredoxin (ABO_0200, not detected), which shuttles electrons to the cytochrome P450 (ABO_0201/2288), an alcohol dehydrogenase (ABO_0202) and a ferredoxin reductase (ABO_0203) (Schneiker *et al.,* 2006). The alcohol dehydrogenase, AlkJ2 (ABO_0202, *P* = 0.008), which converts alcohols generated by the oxidation of an alkane to its corresponding aldehyde, was highly up‐regulated during growth on *n*‐C_14_ and pristane but absent on pyruvate (Fig. 2A). The expression of a ferredoxin reductase (ABO_0203, *P =* 0.049), which oxidizes NAD(P)H to NAD(P)^+^ capturing electrons to transfer to the ferredoxin, was seven‐fold and five‐fold greater during growth on *n*‐C_14_ and pristane, respectively, compared to pyruvate. Multiple alkane monooxygenases were also detected. The alkane 2‐monooxygenase, AlkB2 (ABO_0122) was exclusively expressed only during growth on *n‐*C_14_ (Fig. 2B)_._ The expression of another putative monooxygenase (ABO_2107, *P =* 0.011) was seven‐fold greater on *n‐*C_14_ than pristane with no expression on pyruvate (Fig. 2B). Domain analysis revealed the protein is a member of the FMO (flavin‐binding monooxygenase)‐like family (PF00743), which specializes in the oxidation of xenobiotics. The *rubA* (ABO_0163) *and rubB* (ABO_0162) genes code rubredoxin and rubredoxin reductase, respectively, and are arranged in a putative operon. RubA (ABO_0163), which transfers electrons to the alkane monooxygenase from the rubredoxin reductase, was not detected in this data set and the expression of RubB (ABO_0162), which transfers electrons from NAD(P)H to the rubredoxin, had 5‐fold greater expression on *n*‐C14 compared to pristane (Fig. 2B). Two aldehyde dehydrogenases that catalyse the oxidation of the aldehyde produced from the alcohol dehydrogenase (ABO_0962/ABO_2414) were significantly (P < 0.0001) differentially expressed on *n*‐C_14_ (Fig. 2B). The combination of these alkane monooxygenases or CYP153 cytochrome P450 with an alcohol dehydrogenase and an aldehyde dehydrogenase makes up two alternative pathways for the terminal oxidation of *n*‐alkanes to their corresponding acyl‐CoA derivatives (Fig. 2A and B).

### *Terminal oxidation of the branched alkanes*

**Figure 3:**
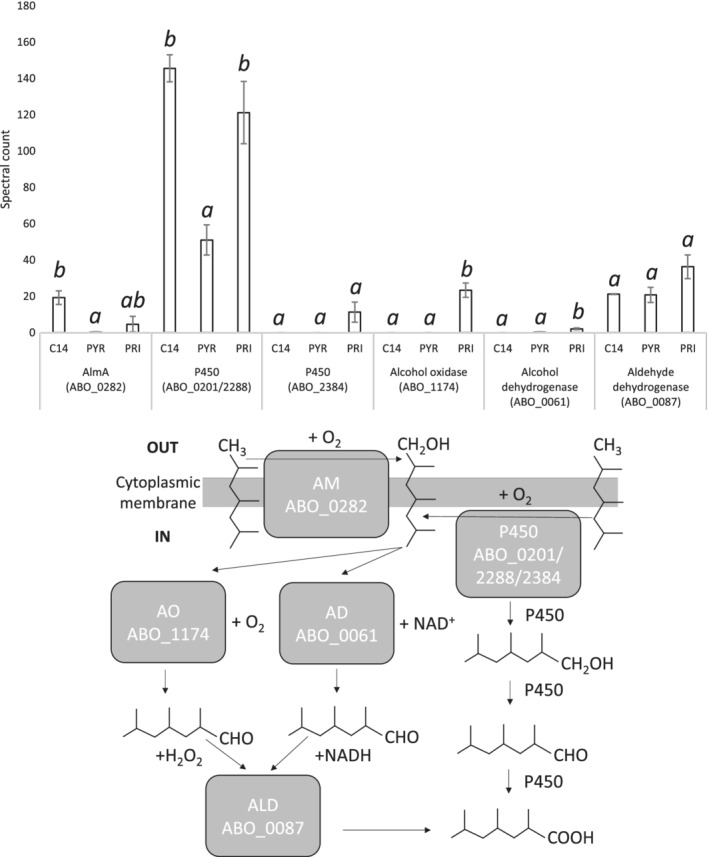
Normalized spectral counts (means ±; *n* = 3) of differentially expressed branched alkane oxidation proteins during growth on a linear alkane (*n*‐C_14_/C14) a non‐hydrocarbon control (Pyruvate/PYR) and a branched alkane (Pristane/PRI) in *A. borkumensis* SK2^T^; treatments not sharing a letter (*a* or *b*) differ at *P* < 0.05 (Tukey's HSD). The alkane monooxygenase (AM) introduces oxygen into the alkane at the terminal carbon converting it into a primary alcohol. This alcohol is further oxidized to an aldehyde by an alcohol oxidase (AO) or alcohol dehydrogenase (AD). An aldehyde dehydrogenase (ALD) converts the aldehyde into a fatty acid, which enters β‐oxidation. Alternatively, a cytochrome P450 (P450) can oxidize the alkane up to it corresponding fatty acid, which enters β‐oxidation.

The expression of an AlmA‐type monooxygenase (ABO_0282) was only present in *n*‐C_14_ and pristane (Fig. 3). It has a 90% identity to the long‐chain flavin‐binding monooxygenase AlmA from *Bacillus halodurans* (AGW21778.1) and a 63% identity to AlmA from *A. dieselolei* B‐5 (B5T_02052). In addition to the cytochrome P450 (ABO_0201/ABO_2288) as previously mentioned, another cytochrome P450 (ABO_2384) was exclusively expressed only when growing on pristane (Fig. 3). A GMC oxidoreductase family protein (ABO_1174, *P* = 0.010) was exclusively expressed during growth on pristane and predicted as an alcohol oxidase in I‐TASSER and TM‐align, which identified an alcohol oxidase from *Pichia pastoris* (5i68A) in PDB as structurally close to ABO_1174 (TM score‐0.822). The FunFam assignment in CATH for matching regions to the ABO_1174 identified the long‐chain‐alcohol oxidase FAO1 (Evalue‐2.5e^−60^). The protein is FAD binding (GO:0050660) suggesting it is an alcohol oxidase, which oxidize primary alcohols into a primary aldehydes and hydrogen peroxide, as they require flavin‐based cofactors, while alcohol dehydrogenases require NAD‐based cofactors. An alcohol dehydrogenase (ABO_0061, *P* = 0.041) was also exclusively expressed during growth on pristane. This suggests *A. borkumensis* can use either and alcohol oxidase or dehydrogenase to convert pristanol to pristanal. No aldehyde dehydrogenase was significantly differentially expressed during growth on pristane, but ABO_0087 was expressed on all substrates tested. The combination of these enzymes makes up a complete pathway for the terminal oxidation of branched alkanes (Fig. 3B).

### *β‐Oxidation of acyl‐CoA derivatives*

**Figure 4:**
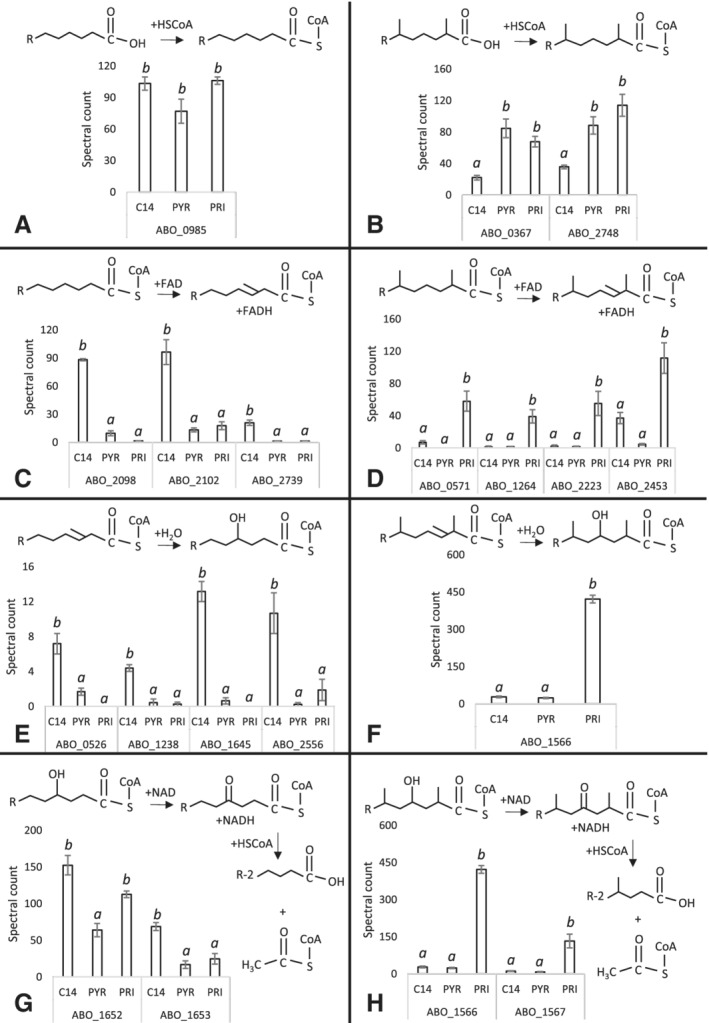
Normalized spectral counts (means ±; *n* = 3) of differentially expressed β‐oxidation proteins during growth on a linear alkane (*n*‐C_14_/C14) a non‐hydrocarbon control (Pyruvate/PYR) and a branched alkane (Pristane/PRI) in *A. borkumensis* SK2^T^; treatments not sharing a letter (*a* or *b*) differ at *P* < 0.05 (Tukey's HSD). The β‐oxidation proteins have putatively been put on either the left‐ or right‐hand side of the figure to represent involvement in straight‐chain alkane degradation (left) or branched‐chain alkane degradation (right) based on their expression pattern. A and B. Acyl CoA synthetases which activate a fatty acid to form acyl‐CoA constitutively expressed all substrates (A) and differentially expressed in pristane (B). C and D. Acyl‐CoA dehydrogenases, which creates a double bond between the second and third carbons down from the CoA group on acyl‐CoA producing trans‐delta 2‐enoyl CoA differentially expressed in *n*‐C14 (C) and pristane (D). E and F. Enoyl CoA hydratases, which remove the double bond in trans‐delta 2‐enoyl CoA adding a hydroxyl group to the to the third carbon down from the CoA group and a hydrogen on the second carbon down from the CoA group producing L‐3‐hydroxyacyl CoA differentially expressed in *n*‐C14 (E) and pristane (F). G and H. The FadAB2 (G) and FadAB (H) operon consisting of β‐oxidation multifunctional enzyme complexes with 3‐hydroxyacyl‐CoA dehydrogenase (removes the hydrogen in the hydroxyl group added to L‐3‐hydroxyacl CoA producing 3‐ketoacyl CoA) and ketoacyl‐CoA thiolase (attaches a CoA group on the third carbon down from the CoA group producing acetyl‐CoA and acyl‐CoA which is two carbons shorter) activities were differentially expressed on *n*‐C_14_ and pristane, respectively.

The fatty acids generated by oxidation of *n‐*alkanes are further metabolized by β‐oxidation. The β‐oxidation proteins detected have putatively been assigned functions for their potential role in straight‐chain alkane degradation (Fig. 4 A, C, E and G, left side) or branched‐chain alkane degradation (Fig. 4B, D, F and H, right side) based on their pattern of expression. Before a fatty acid can enter any metabolic pathway, it must first be activated to form acyl‐CoA. This reaction is catalysed by acyl‐CoA synthetases, generating AMP. No acyl‐CoA synthetase was differentially expressed on *n‐*C_14_ compared to pristane. ABO_0985 was initially annotated as an acid thiol ligase but a BLASTP search found it is identical (100% identity) to long‐chain acyl CoA synthetases in multiple *Alcanivorax* sp. (97CO‐5; 97CO‐6; NBRC 101098). This protein was expressed equally across the three growth substrates with high spectral counts (>75, Fig. 4A) and could potentially be constitutively expressed, suggesting it can potentially use branched and linear fatty acids as substrates. Two acyl‐CoA synthetases potentially catalysing this reaction, fadD/ABO_0367 (*P* = 0.041) and alkK/ABO_2748 (*P* = 0.046) were differentially expressed on pristane compared to *n‐*C_14_ but also similarly expressed on pyruvate (Fig. 4B). Acyl‐CoA dehydrogenase creates a double bond between the second and third carbons down from the CoA group on acyl‐CoA producing trans‐delta 2‐enoyl CoA and in the process, uses FAD as an electron acceptor reducing it to FADH_2_. Four acyl‐CoA dehydrogenases were significantly differentially expressed during growth on pristane, including ABO_0571 (*P* = 0.024), ABO_1264 (*P* = 0.023), acdA/ABO_2223 (*P* = 0.046) and ABO_2453 (*P* = 0.017) (Fig. 4D), while three other acyl‐CoA dehydrogenases were significantly differentially expressed during growth on *n*‐C_14_ (Fig. 4C). These include fadE/ABO_2098 (*P* < 0.0001), ABO_2102 (*P* = 0.004) and ABO_2739 (*P* = 0.001). Enoyl CoA hydratase removes the double bond in trans‐delta 2‐enoyl CoA adding a hydroxyl group to the third carbon down from the CoA group and hydrogen on the second carbon down from the CoA group producing L‐3‐hydroxyacyl CoA. Four enoyl CoA hydratases were significantly differentially expressed during growth on *n*‐C_14_ compared to pristane (Fig. 4E), including/ABO_0526 (*P* = 0.005), ABO_1238 (*P* = 0.006), fadC/ABO_1645 (*P* < 0.001) and eno/ABO_2556 (*P* = 0.020). No specific enoyl CoA hydratases were differentially expressed on pristane. However, *fadB* (ABO_1566, *P* < 0.0001), which was up‐regulated on pristane, from the *fadAB* operon codes the α‐subunit of a β‐oxidation multifunctional enzyme complex possessing both 3‐hydroxyacyl‐CoA dehydrogenase and enoyl‐CoA hydratase activities (Fig. 4F). 3‐hydroxyacyl CoA dehydrogenase removes the hydrogen in the hydroxyl group added to L‐3‐hydroxyacl CoA producing 3‐ketoacyl CoA, using NAD as an electron acceptor reducing it to NADH. In the final step of β‐oxidation ketoacyl‐CoA thiolase attaches a CoA group on the third carbon down from the CoA group resulting in the formation of two molecules, an acetyl‐CoA and an acyl‐CoA that is two carbons shorter. Only one ketoacyl‐CoA thiolase was detected (ABO_2452, *P* = 0.446), which was not expressed on pyruvate but was not significantly differentially expressed on either *n*‐C_14_ and pristane due to detection of very low spectral counts. The *fadAB* operon consisting of *fadB* (ABO_1566, *P* < 0.0001) previously mentioned above and *fadA* (ABO_1567, *P* = 0.024), which codes for the β‐subunit of the complex with 3‐ketoacyl‐CoA thiolase activity, was significantly differentially expressed on pristane (Fig. 4H). The *fadAB2* operon consisting of *fadB2* (ABO_1652, *P* = 0.011), which was differentially expressed on both *n*‐C_14_ and pristane, and *fadA* (ABO_1653, *P* = 0.022), which was significantly differentially expressed on *n*‐C_14_ (Fig. 4G).

## Discussion

### *Terminal oxidation of linear* n*‐alkanes*

Bacteria degrading linear alkanes typically contain a non‐heme alkane oxygenase system including a membrane bound alkane monooxygenase, a rubredoxin and a rubredoxin reductase and/or a heme‐thiolate cytochrome system including a soluble cytochrome P450, a ferredoxin and ferredoxin reductase (Teimoori *et al.,* 2011; Nie *et al.,* 2014). It is rather common to find bacteria that contain more than one alkane oxidation system as each have different substrate ranges or different induction patterns. *A. borkumensis* has two alkane monooxygenases on its genome, which are homologous to well characterized the alkane hydroxylase AlkB from *Psudomonas putida* GPo1, which oxidizes alkanes from *n*‐C_5_ to *n*‐C_16_ (Schneiker *et al.,* 2006; van Beilen and Funhoff, 2007). AlkB_1_ (ABO_2707) and AlkB_2_ (ABO_0122) oxidizes alkanes in the range of *n*‐C_5_ to *n*‐C_12_ and *n*‐C_8_ to *n*‐C_16_, respectively (Hara *et al.,* 2004; van Beilen *et al.,* 2004). AlkB_2_ was exclusively expressed during growth on *n*‐C_14._ Electrons are transferred to the AlkB active site via a rubredoxin. There are two rubredoxin genes, *alkG* (ABO_2708) and *rubA* (ABO_0163) in the *A. borkumensis* genome (Schneiker *et al.,* 2006). Both rubredoxins were however not detected in this data set. Rubredoxin reductases oxidizes NAD(P)H to transfer electrons to rubredoxin reducing it allowing electron transfer to the monooxygenase. There is one rubredoxin reductase gene in the *A. borkumensis* genome, *RubB* (ABO_0162), which has been shown to reduce AlkG when both proteins were cloned and functionally overexpressed in *E. coli* (Teimoori *et al.,* 2011). This activity compensates for a lack of AlkT, also a rubredoxin reductase, missing in the *A. borkumensis* genome (Schneiker *et al.,* 2006). RubB expression was greatest during growth on *n*‐C_14,_ although expression was observed while growing on all three substrates at levels that were not significantly different. *alkB1/alkB2* expression is strictly *n*‐alkane dependent, as confirmed here by the expression of AlkB2 only while growing on *n*‐C_14_, but RubA/RubB are constitutively expressed in other bacteria (Geissdörfer *et al.,* 1999; Tani *et al.,* 2001; Marín *et al*., 2003). The unbalanced expression between monooxygenase (AlkB2) to rubredoxin/rubredoxin reductase (RubA/RubB) suggest that the molecules of membrane‐bound monooxygenases share a limiting number of rubredoxin and rubredoxin reductase molecules, which are soluble proteins that can probably associate and dissociate from the membrane‐bound monooxygenase, as seen in *P. aeruginosa* (Marín *et al*., 2003).

*A. borkumensis* contains alternative enzyme systems to AlkB for terminal oxidation of alkanes (Figs. 2 and 3), including three CYP153 cytochrome P450s; ABO_0201 (P450‐1/P450‐c), ABO_2288 (P450‐2/P450‐b) and ABO_2384 (P450‐3) (Schneiker *et al.,* 2006). The expression of ABO_0201 and ABO_2288 was higher on *n*‐C_14_ than pyruvate, suggesting it can terminally oxidize medium‐chain alkanes. ABO_2384 was not expressed on *n‐*C_14_ suggesting it may act on different substrates. A similar trend has been observed in other bacteria with multiple CYP153s. For example, *Sphingomonas* sp. HXN200 possesses five CYP153 genes, three of which show activity towards *n‐*C_5_‐*n*‐C_10_, whereas no affinity for these substrates was observed for the other two genes, suggesting that these genes are either pseudogenes or act on different substrates (Van Bogaert *et al.,* 2011). P450‐1 and P450‐2 are phylogenetically grouped in one branch with CYP153A1 from *Acinetobacter* sp. EB104, which was experimentally confirmed to catalyse terminal alkane oxidation (Maier *et al.,* 2001; Schneiker *et al.,* 2006; van Beilen *et al.,* 2006). The coexpression of AlkB and CYP153 may cooperatively ensure that *A. borkumensis* can utilize a broader range of linear substrates.

The expression of an AlmA homologue (ABO_0282) was at least 4‐fold greater during growth on *n*‐C_14_ differentially expressed compared to pyruvate (Fig. 2). Although AlmA is a long‐chain monooxygenase, it has been shown in other bacteria to have some activity on shorter alkanes but has much lower activity compared to growth on long‐chain alkanes, for example, >*n*‐C_20_. For example, *almA* gene expression of *M. koreensis, M. alkaliphilus* and *M. lipolyticus* responded to alkanes ranging in length from *n‐*C_9_ up to *n*‐C_36_ (Wang and Shao, 2012a). AlmA in *A. dieselolei* was shown to have activity against alkanes ranging in length from *n‐*C_10_ up to *n*‐C_36_. This suggests that *n‐*C_14_ is within the substrate range of ABO_0282 and therefore the protein is expressed. However, if *A. borkumensis* was grown on longer alkanes the expression of ABO_0282 would potentially be much higher.

### *Terminal oxidation of branched alkanes*

The reason branched alkanes are harder to degrade may be either that the alkyl branches hinder the uptake of the hydrocarbons into the cell or that the branches are not susceptible to the enzymes of the β‐oxidation pathway (Schaeffer *et al.,* 1979). One of the conditions necessary for aerobic degradation of branched hydrocarbons in nature is that the bacteria must possess genes and enzymes for initial oxygenation (Britton, 1984; Beguin *et al.,* 2003). The degradation pathways of pristane have been established based on the analyses of the metabolic intermediates formed from pristane using *Brevibacterium erythrogenes* (Pirnik *et al.,* 1974), *Nocardia globerula* (Alvarez *et al.,* 2001), and several strains such as *Corynebacterium, Mycobacterium* and *Nocardia* (McKenna and Kallio, 1971; Pirnik *et al.,* 1974; Beguin *et al.,* 2003). Pristane is degraded by at least three catabolic pathways: monoterminal, diterminal and subterminal oxidation (Nhi‐Cong *et al.,* 2009). The metabolites produced from monoterminal oxidation of pristane are pristanol, pristyl aldehyde and pristanic acid (Nakajima *et al.,* 1985).

The initial hydroxylation reaction converting pristane to pristanol could be catalysed by the AlmA homologue (ABO_0282), P450‐1/2 (ABO_0201/2288) and/or P450‐3 (ABO_2384) (Fig. 3). AlmA expression was significantly higher on pristane compared to pyruvate, but the expression was highest on *n*‐C_14_. The expression of P450‐1/2 was at least 2‐fold higher on both *n*‐C_14_ and pristane compared to pyruvate. P450‐3 was exclusively expressed on pristane. AlmA has previously been implicated in pristane degradation in other *Alcanivorax* strains. In *A. dieselolei* B‐5 pristane activated the expression of AlmA (Liu *et al.,* 2011). Enzymatic assays showed it converted pristane to its corresponding primary alcohol (Wang and Shao, 2014). In *A. hongdengensis* A‐11‐3 pristane selectively activated the expression *almA* (Wang and Shao, 2012b). In other OHCB expression of AlmA was enhanced in four *Marinobacter* strains (*M. koreensis* L53‐1‐2, *M. alkaliphilus* L53‐10‐4, *M. alkaliphlius* L52‐11‐18 and *M. lipolyticus* 342‐2) when grown in the presence of pristane (Wang and Shao, 2012a). P450‐1 from *A. borkumensis* is expressed from the promoter of the upstream gene (ABO_0199), and this promoter was more active when *n‐*C_8_‐*n*‐C_18_ or pristane were assimilated than when pyruvate was available (Sevilla *et al.,* 2017). This is consistent with our results as P450‐1 is differentially expressed in *n*‐C_14_ and pristane compared to pyruvate suggesting it can use either linear or branched alkanes as substrates. P450‐3 is phylogenetically distant from the other two cytochromes in *A. borkumensis* suggesting it may have a different substrate specificity, that is, branched alkanes (Wang and Shao 2012b; Sevilla *et al.,* 2017). Further evidence of this has been shown by Schneiker *et al*. (2006), who confirmed that the branched alkane phytane strongly induce the expression of P450‐3 in *A. borkumensis* SK2^T^. In *A. hongdengensis* A‐11‐3 pristane selectively activates the expression of P450‐3 (Wang and Shao, 2012b), and the expression of P450‐3 in *A. borkumensis* was much higher when cells assimilated pristane in contrast to when *n*‐alkanes or pyruvate were available (Sevilla *et al.,* 2017). This was confirmed in our study as P450‐3 was exclusively expressed on pristane.

The conversion of pristanol to pristyl aldehyde could be catalysed by either the alcohol oxidase (ABO_1174; exclusively expressed on pristane) or alcohol dehydrogenase (ABO_0061; two‐fold up‐regulated on pristane), which utilize two different biological modes of alcohol dehydrogenation (Fig. 3). Alcohol dehydrogenases are nicotinamide dependent, while alcohol oxidases are flavin dependent and generate hydrogen peroxide (Geissler and Hemmerich, 1981; Kemp *et al.,* 1988). Following terminal oxidation, the alcohols generated are normally oxidized to the corresponding aldehyde by means of alcohol dehydrogenases in bacteria, although alcohol oxidases have been reported in alkane degrading yeasts and moulds, for example, *Candida* spp. where they functionally substitute alcohol dehydrogenases (Blasig *et al.,* 1988; Kemp *et al.,* 1988; Hommel and Ratledge, 1990).

For the conversion of pristyl aldehyde to pristanic acid no aldehyde dehydrogenases were significantly differentially expressed during growth on pristane. An aldehyde dehydrogenase (ABO_0087) was expressed on all substrates tested meaning it may be constitutively expressed. Also, catalysis of sequential oxidation reactions is usual in cytochrome P450 reactions particularly with xenobiotics (Guengerich *et al.,* 2011) (Fig. 3). P450 enzymes catalyse a notably diverse range of oxidative transformations on a very wide range of substrates (Meunier *et al.,* 2004). P450 enzymes can oxidize aldehyde to carboxylic acids through a well understood mechanism (Newcomb *et al.,* 2003) and examples include the oxidation of aliphatic α,β‐unsaturated aldehydes and anthraaldehyde to the corresponding acids by the latter enzyme (Guengerich *et al.,* 2011). This means the cytochrome P450 could potentially functionally substitute an aldehyde dehydrogenase generating a fatty acid, which is further degraded through β‐oxidation.

## β‐oxidation

### *β‐Oxidation of acyl‐coA derivatives*

*A. borkumensis* expressed a different set of enzymes for β‐oxidation depending on the growth substrate (branched vs. linear alkane) utilized (Fig. 4). Fatty acids generated from the oxidation of alkanes are transported through the cytoplasmic membrane coupled with the activation to acyl‐CoA thioesters catalysed by the acyl‐CoA synthetase with concomitant hydrolysis of ATP (Fiedler *et al.,* 2002). Acyl‐CoA synthetases are widely distributed in both prokaryotic and eukaryotic organisms and exhibit a broad substrate specificity (Ruth *et al.,* 2008). ABO_0367 encodes a fadD homologue (Fig. 4B). The product of the *fadD* gene is a long‐chain fatty acyl‐CoA ligase that converts exogenous long‐chain fatty acids (LCFAs) into acyl‐CoA thioesters when they are transported in bacteria (Black *et al.,* 1992). The mechanism behind FadD substrate specificity is not fully understood. Structures of a homologous long chain acyl‐CoA synthetase from *Thermus thermophilus* and a medium chain acyl‐CoA synthetase from *Homo sapiens* suggest that it is the length of the FadD fatty acid binding pocket that determines specificity (Hisanaga *et al.,* 2004; Kochan *et al.,* 2009). A consensus sequence (^1^DGWLHTGDIG*X*W*X*P*X*G*X*LKIIDRKK^25^) is common to all fatty acyl‐CoA synthetases and represents a FACS signature motif (Black *et al.,* 1997). Mutational studies revealed subtle changes in this region result in marked changes with respect to substrate recognition (e.g. D^22^ and K^25^) defining the fatty acid specificity of the enzyme (Black *et al.,* 1997). For example, fatty acyl‐CoA synthetases from *Pseudomonas oleovorans* and *Saccharomyces cerevisiae*, which lack the lysyl residue at the position equivalent to K^24^ have specificity to medium‐chain fatty acids (van Beilen *et al.,* 1992; Knoll *et al.,* 1994). ABO_0367 (three‐fold higher expression on pristane compared to *n*‐C_14_) has a valine residue in the K^24^ position and a glycine in the D^22^ in the FACS signature motif, which may contribute to its preference to branched fatty acids over straight‐chain fatty acids. ABO_2748, which had 3‐fold higher expression on pristane compared to *n*‐C_14_, is an AlkK homologue (Fig. 4B). The gene product of *alkK* in *Psudomonas putida* encodes a medium‐chain acyl CoA synthetase, which catalyses the final reaction in alkane oxidation, converting medium‐chain length fatty acid to their CoA derivatives (van Beilen *et al.,* 1992). The enzyme was able to complement a fadD mutation in *E. coli* K‐12 verifying its predicted role (van Beilen *et al.,* 1992). Assays using branched hydroxyalkanoates as substrates confirmed AlkK is also capable of converting branched substrates to their corresponding acyl‐CoA thioesters (Satoh *et al.,* 2005). As AlkK is capable of utilizing branched substrates this may account for the higher spectral counts during growth on pristane.

The *A. borkumensis* genome contains two *fadAB* operons, consisting of β‐oxidation multifunctional enzyme complexes with 3‐hydroxyacyl‐CoA dehydrogenase and ketoacyl‐CoA thiolase activities that were detected in our data set (Fig. 4G and H). They exhibited different expression patterns depending on the substrate utilized (FadAB/ABO_1566/1567 was expressed on the branched pristane, and FadAB2/ABO_1652/1653 was expressed on the linear alkane). It has been widely reported that different FadAB isoforms have different substrate specificities and multiple FadAB operons exist within a microbes genome. For example, *Escherichia coli* possess an anaerobic paralog of the canonical FadAB, YfcYX (Campbell *et al.,* 2003). There are multiple sets of FadAB complexes described in the model strain *Pseudomonas putida* KT2440, which accounts for the huge metabolic versatility of this strain (Nelson *et al.,* 2002; Ouyang *et al.,* 2007). More restricted substrate specificity was reported for *Mycobacterium tuberculosis* FadB, which needs the action of a trans‐acting enoyl‐CoA isomerase in order to completely degrade cis‐unsaturated fatty acids (Srivastava *et al.,* 2015). In the case of *Streptomyces coelicolor* FadAB complexes, low efficiency or narrow substrate specificity range of the complexes accounted for lower growth rates exhibited by two of the three complexes during growth on oleic acid as the sole carbon source. (Menendez‐Bravo *et al.,* 2017).

## Conclusions

In conclusion, *A. borkumensis* SK2^T^, an important OHCB that can dominate microbial communities following marine oil spills, possesses the ability to degrade branched alkanes, which provides it a competitive advantage over many other marine alkane degraders that can only degrade linear alkanes. This study has significantly enhanced our understanding of the fundamental physiology of *A. borkumensis* SK2^T^ by the identification of the expressed enzymes for degrading linear and branched alkanes. It has also highlighted the differential expression of sets of β‐oxidation proteins to overcome steric hinderance from branched substrates.

## Experimental procedures

### *LC–MS/MS shotgun analysis of* A. borkumensis *SK2^T^*

#### *Culture conditions*

*A. borkumensis* SK2^T^ (DSM 11573) was grown from a frozen stock in sterile 160 ml Nunc Cell Culture‐Treated Flasks containing 100 ml of ONR7a media (Dyksterhouse *et al.,* 1995). Cultures were supplemented with tetradecane (*n*‐C_14_), pristane or pyruvate as the sole carbon source. The starting inoculum was 20 μl of exponentially growing cells (from a 3 day old pre‐cultures of cells growing on either *n*‐C_14_, pristane or pyruvate that were in the exponential growth phase, i.e., the first measurable day of growth by OD). Cultures were incubated in an orbital shaker (16°C, 60 rpm). Growth curves were highly similar on all three substrates, and cells were harvested for protein extraction after 4 days, which represented cells in early exponential growth phase (determined by growth curves measured by OD at 600 nm, that showed a two day lag phase, followed by exponential growth measurable by an increase in OD from day 3, with cells entering stationary phase after 14 days).

#### *LC–MS/MS proteomics*

Cells were harvested from 50 ml of each culture by centrifugation (4600 × g, 15 min) and washed in 2 ml of phosphate buffered saline. Total protein was immediately extracted by resuspending the cell pellet in 75 μl of extraction buffer (62.5 mM TRIS–HCl pH 6.8, 10% glycerol w/v, 12 mM dithiothreitol (DTT), 2% sodium dodecyl sulfate (SDS) v/v and one Pierce Protease Inhibitor Tablet per 50 ml), heating in a water bath (95°C, 12 min) and then centrifuging (10,500 × g, 5 min) to remove cell debris. Proteins extracts were visualized by SDS‐PAGE and trypsin digestion, and LC–MS/MS with a ThermoFisher hybrid high‐resolution LTQ Orbitrap instrument was performed as previously described (McKew *et al.,* 2013).

#### *MS/MS analysis*

MS/MS analysis was performed in MaxQuant (Cox and Mann, 2008). The LTQ Orbitrap raw data files were first converted to MSM files with the MaxQuant ‘Quant’ module. The open‐source search engine Andromeda, which is integrated into MaxQuant, was used to identify peptides in sequence databases by their fragmentation spectra (Cox *et al.,* 2011). Peptides and proteins were filtered at 0.3% false discovery rate (FDR) to obtain the final data sets. Proteins were quantified by counting the number of MS/MS spectra matched to the corresponding proteins. Uniprot protein sequences from the *A.borkumensis* SK2^T^ genome (Schneiker *et al.,* 2006) were used to perform protein identification. Proteins were validated using the default settings in MaxQuant and Andromeda with a minimum of at least one peptide, but that any such protein had to unambiguously identified by peptides that were unique to that protein. Spectral counts were normalized to total spectral counts that account for differences between runs (total spectral counts varied between 11,015 and 15,856 per run).

#### *Statistical and bioinformatic analysis*

Differential expression analysis was performed by analysis of variance (ANOVA) and Tukey's HSD test with Benjamini‐Hochberg post‐hoc corrections (Benjamini and Hochberg, 1995) within the XLSTAT‐Premium Version 2016.1 (Addinsoft) ‘OMICs’ package. All proteins significantly (*P* < 0.05) up‐regulated during growth on hydrocarbons were subjected to a BLAST (Basic Local Alignment Search Tool) (Altschul *et al.,* 1990) against the NCBI nr database. Protein family and domain analysis was carried out in Pfam v30.0 (Finn *et al.,* 2016). Proteins were assigned to functional families by hierarchical classification of protein domains based on their folding patterns in CATH v4.1 (Class, Architecture, Topology, Homology) (Sillitoe *et al.,* 2015). Full length secondary and tertiary structure predictions, functional annotations on ligand‐binding sites, enzyme commission numbers and gene ontology terms were generated using the I‐TASSER SERVER (Zhang, 2008).

## Supporting information


**Table S1** Mean spectral counts (*n* = 3) of detected proteins during growth on a linear alkane (*n*‐C_14_), a branched alkane (Pristane) and a non‐hydrocarbon control (Pyruvate) in *Alcanivorax borkumensis* SK2^T^, with differential expression analysis (Anova with Benjamini Hochberg FDR correction (P‐value) and Tukey HSD Post Hoc Test (a,b,c in parentheses indicate significant differences by Tukey HSD test)
